# Che1/AATF interacts with subunits of the histone acetyltransferase core module of SAGA complexes

**DOI:** 10.1371/journal.pone.0189193

**Published:** 2017-12-12

**Authors:** Gizem Caliskan, Ikbal C. Baris, Ferhan Ayaydin, Melanie J. Dobson, Muge Senarisoy, Imre M. Boros, Zeki Topcu, Sevil Zencir

**Affiliations:** 1 Department of Pharmaceutical Biotechnology, Faculty of Pharmacy, Ege University, Izmir, Turkey; 2 Department of Medical Biology, Faculty of Medicine, Pamukkale University, Denizli, Turkey; 3 Cellular Imaging Laboratory, Biological Research Center, Hungarian Academy of Sciences, Szeged, Hungary; 4 Department of Biochemistry & Molecular Biology, Dalhousie University, Halifax, NS, Canada; 5 Institute of Biochemistry, Hungarian Academy of Sciences, Szeged, Hungary; Institute of Genetics and Molecular and Cellular Biology, FRANCE

## Abstract

General Control Non-derepressible 5 (GCN5) and Alteration/Deficiency in Activation 2 and 3 proteins (ADA2 and ADA3, respectively) are subunits of the Histone AcetylTransferase (HAT) module of SAGA- and ATAC-type co-activators. We previously reported four new interacting partners of human ADA3 identified by screening a human fetal brain cDNA library using yeast two hybrid technology. One of these partners was Apoptosis-Antagonizing Transcription Factor (AATF), also known as Che-1, an RNA polymerase II-binding protein with a number of roles in different cellular processes including regulation of transcription, cell proliferation, cell cycle control, DNA damage responses and apoptosis. Che-1/AATF is a potential therapeutic target for cancer treatments. In this study, we aimed to identify whether besides ADA3, other components of the HAT modules of SAGA and ATAC complexes, human ADA2 and GCN5 also interact with Che-1/AATF. Co-immunoprecipitation and co-localization experiments were used to demonstrate association of AATF both with two ADA2 isoforms, ADA2A and ADA2B and with GCN5 proteins in human cells and yeast two-hybrid assays to delineate domains in the ADA2 and GCN5 proteins required for these interactions. These findings provide new insights into the pathways regulated by ADA-containing protein complexes.

## Introduction

The compact structure of chromatin in eukaryotic cells has an impact on all nuclear processes that require DNA [1,2]. Among these transcriptional regulation is also tightly associated with chromatin dynamics. Activation requires modification of the nucleosome architecture by chromatin-remodeling and histone-modifying complexes. The histone tails protruding from the nucleosomes are subjected to various post-translational alterations [3]. Acetylation of histones modifies the physical and chemical properties of the chromatin and is one of the most studied modifications executed by histone acetyltransferase (HAT) complexes. The evolutionarily well-conserved HAT enzyme GCN5 specifically targets histones H3 and H2B as part of different HAT complexes with different sizes and important roles in transcriptional regulation [1–3]. GCN5 is part of several highly conserved multiprotein coactivator complexes, known as SAGA (Spt-Ada-Gcn5 Acetyltransferase) and ADA complexes in yeast, and as SAGA and ATAC (Ada-Two-A-Containing) complexes in metazoans [4–7]. These complexes consist of modules with specific functions, which might include HAT, deubiquitination, structural as well as activator interaction modules, with each built from specific and/or shared subunits [5]. The HAT module of ATAC is composed of the catalytic subunit GCN5, ADA2A, ADA3, and SGF29, whereas the HAT module of metazoan SAGA contains ADA2B, instead of ADA2A [5]. Besides HAT activity, GCN5 is also known to display protein acetyltransferase activity and is known to be effective in cellular response under stress conditions [8].

In addition to ADA family members, several other proteins such as TATA-binding protein-(TBP)-associated factors (TAFs) and Spt proteins are components of the GCN5-containing complexes, i.e., SAGA of yeast, or the related STAGA/TFTC (SPT3-TAFII31-GCN5L-acetylase)/(TATA-binding protein-free TAF-containing complex) in humans, as well as the metazoan-specific ATAC [4–7]. Although the exact mechanism of the regulation by the individual components of these multiprotein complexes is yet to be clarified, a large body of evidence indicates that their function is fulfilled through multi-protein interactions.

We previously reported four novel partners of ADA3 identified by screening a human fetal brain cDNA library using yeast two-hybrid (Y2H) technology [9]. One of these partners was Apoptosis-Antagonizing Transcription Factor (AATF), also known as Che-1, an RNA polymerase II-specific transcription factor that participates in the regulation of gene expression by mediating physical interactions between sequence-specific activators and transcription machinery [10–12]. Che-1/AATF is a 560 aa protein with N-terminal acidic domain, a canonical leucine zipper and three LXXLL motifs [10]. Among the roles attributed to AATF are involvement in cell cycle control and DNA damage response (DDR) through activation of p53 [11,13–16]. AATF overexpression and depletion are associated with tumor suppression and drug sensitivity [11,14,15,17,18].

ADA2, ADA3 and GCN5 act as heterotrimer and the interaction of GCN5 with the other subunits of the HAT module modulates its activity and specificity. We hypothesized that similarly to ADA3, the other subunits of GCN5-containing HAT modules, ADA2 and GCN5 might also associate with AATF. In this study co-immunoprecipitation (Co-IP) and co-localization of fluorescently-tagged proteins provided support for AATF interacting with ADA2A, ADA2B and GCN5 in human cells and Y2H assays were used to identify the AATF-interacting regions in the ADA2 and GCN5 proteins.

## Results

### ADA2 and GCN5 proteins, but not SGF29, interact with AATF

**Fig 1:**
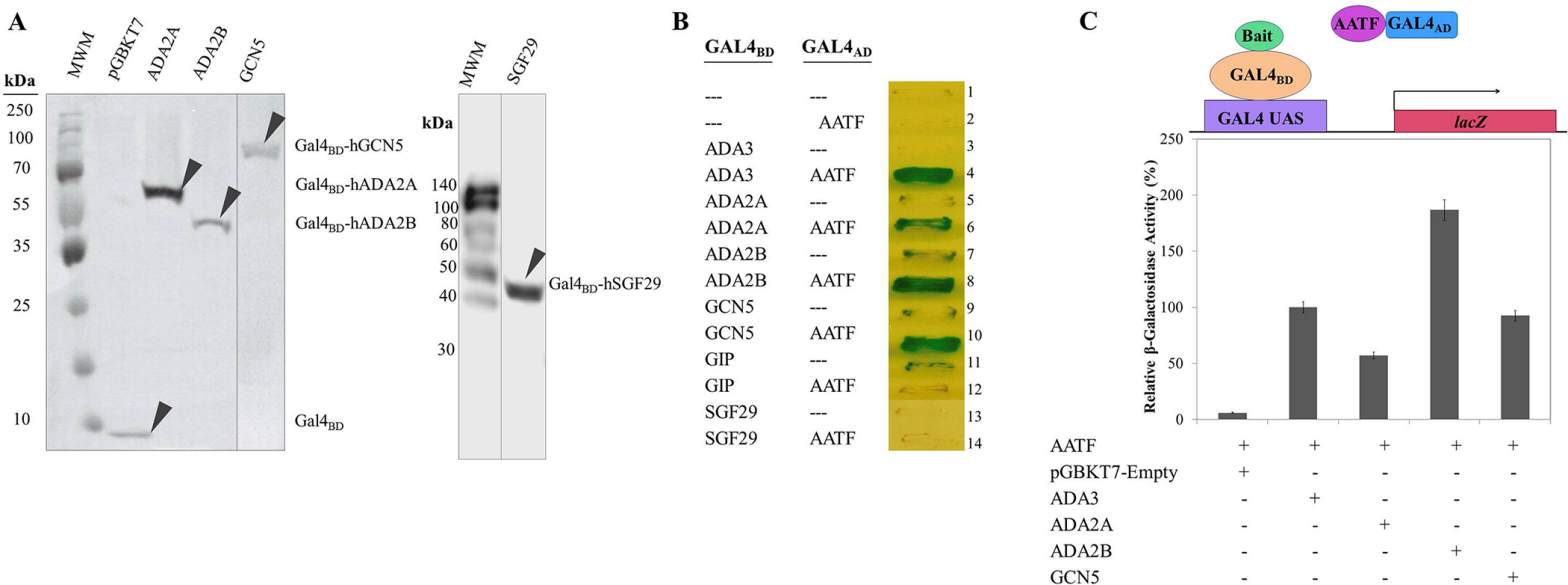
AATF interacts with ADA2A, ADA2B and GCN5 but not with SGF29 in a yeast two-hybrid assay. Reporter gene activities resulting from the interaction of Gal4_BD_-hADA2A, -ADA2B, -GCN5 and Gal4_AD_-AATF fusion proteins were determined with qualitative and quantitative assays. (A) A yeast two-hybrid reporter strain was transformed with plasmids that expressed human ADA2A, ADA2B, GCN5 or SGF29 as Gal4_BD_-fused proteins (pGBKT7-ADA2A, pGBKT7-ADA2B, pGBKT7-GCN5, and pGBKT7-SGF29, fusion proteins of ~60 kDa, 55 kDa, 104 kDa, and 33 kDa, respectively) or empty vector (pGBKT7). Total yeast cell extracts were separated by 10% SDS-PAGE and analysed by immunobloting with antibodies specific for Gal4_BD_. (MWM: molecular weight marker. Bands representing Gal4_BD_ and Gal4_BD_ fusion proteins are indicated with arrowheads). (B) Detection of reporter gene activity resulting from the interaction of Gal4_AD_-AATF fusion protein with Gal4_BD_ fusion proteins. Yeast (strain AH109) cells were co-transformed with plasmids expressing Gal4_BD_- and Gal4_AD_-fusion proteins as indicated. Co-transformants were streaked on solid selective medium (SD/-Ade/-His/-Trp/-Leu/X-α-Gal) and incubated for 3 days at 28°C. Growth and expression of the alpha-galactosidase reporter gene (indicated by the dark colour) shows the interaction of the two hybrid proteins. The Gal4_BD_ fusion proteins, ADA3 and GIP were used as positive and negative controls, respectively. Vector with no insert is indicated by triple hyphen-minus (—). (C) Results of quantitative assay for interactions. β-gal enzyme activity encoded by *lacZ* gene was detected via calorimetric assay and expressed as percentage relative to activity resulting from the interaction of AATF and ADA3. Results represent the average ± SD from three independent assays. β-gal activity was significantly increased when AATF was co-expressed with ADA3, ADA2A, ADA2B or GCN5 in comparison to control plasmid with no insert (ANOVA, *p* < 0.01).

Human ADA2 has two isoforms, ADA2A and ADA2B [5,19,20]. Since the two isoforms are found in distinct HAT complexes with different size and subunit compositions, we included both ADA2A and ADA2B variants in our investigation because their differences could, in turn, reveal different interactions. Interaction with SGF29, a protein component that distinguishes SAGA-related complexes from ADA complexes [5–7] was also assessed. We constructed plasmids that would express the ADA2s, GCN5 and SGF29 proteins in yeast fused to the DNA-binding domain of the yeast transcription factor Gal4 (Gal4_BD_), verified their sequence and showed the expression of the expected Gal4_BD_-fusion proteins in yeast by western blotting. Each construct expressed the corresponding protein with anticipated molecular weight (Fig 1A). Expression was not toxic in yeast as indicated by the comparable number and size of the transformed yeast colonies obtained with vector relative to plasmids expressing the fusion proteins. Moreover, ADA2s, GCN5 and SGF29 did not activate transcription of the Gal4-responsive *ADE2* or *HIS3* reporter genes in the absence of an interacting protein fused to the Gal4 transcription activation domain (Gal4_AD_) (Fig 1B).

Next we examined the individual interactions between the Gal4_BD_-fused constructs (pGBKT7-ADA2A, -ADA2B, -GCN5, and-SGF29) by co-expressing them with Gal4_AD_-fused AATF (pGADT7-AATF) (Fig 1B). The vectors pGBKT7 and pGADT7 were included as a negative control pair to compare the transcriptional activation of reporter genes in yeast while pGBKT7-ADA3 was used as positive control (Fig 1B). While ADA2A, ADA2B and GCN5 manifested strong interactions upon co-expression with the AATF fusion protein (Fig 1B, lanes 6, 8 and 10, respectively), another plasmids expressing Gal4_BD_ fused to the human Glutaminase Interacting Protein (pGBKT7-GIP), known to have different localization and function [21,22], or to SGF29 did not reveal any interaction with AATF (Fig 1B, lanes 12 and 14), which also provides support for the specificity of the interactions to ADA and GCN5 proteins.

The interaction between the fusion proteins in the yeast was quantified by measuring β-galactosidase enzyme activity expressed by the Gal4-responsive *lacZ* gene in the two-hybrid reporter yeast strain [23,24]. ADA2B produced the strongest interaction with AATF, while ADA2A and GCN5 displayed weaker, nonetheless definite interaction, comparable to that detected between ADA3 and AATF. Our one-way analysis of variance (ANOVA) analysis showed that β-galactosidase activity of the *lacZ* promoter significantly increased in co-transformations of the ADA3, ADA2s and GCN5 with AATF compared to co-transformation with the control plasmid without insert (*p* <0.01).

### Identification of domains of ADA2A, ADA2B and GCN5 required for AATF interaction

**Fig 2:**
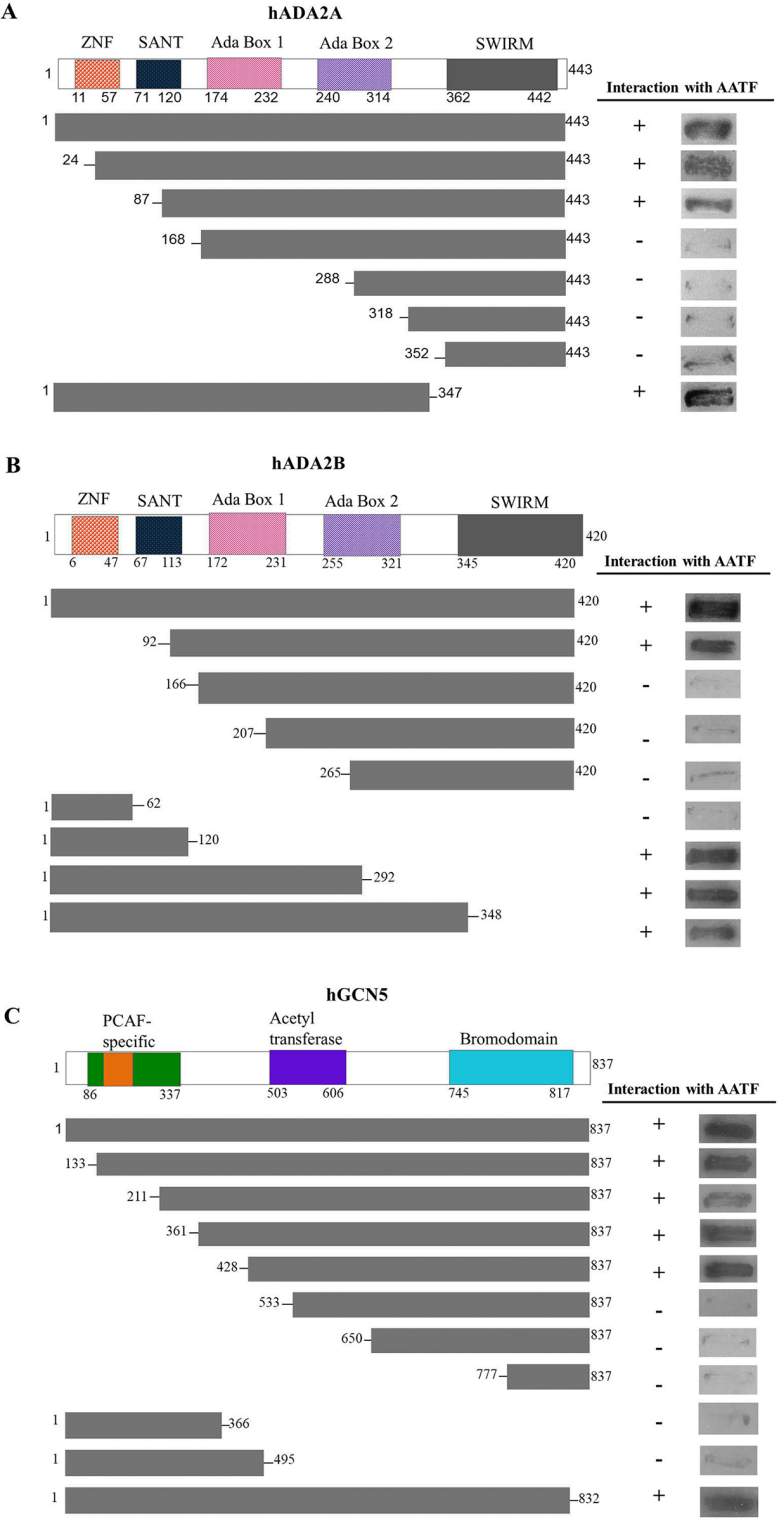
Mapping the regions of ADA2A, ADA2B and GCN5 required for interactions with AATF. AH109 yeast cells were co-transformed with a plasmid expressing either full length or selected regions of ADA2A (A), ADA2B (B) and GCN5 (C) as a Gal4_BD_ fusion proteins and with a second plasmid expressing AATF fused to Gal4_AD_. Growth on the selective medium (SD/-Ade/-His/-Leu/-Trp/X-α-gal) and dark colour due to expression of the α-galactosidase reporter gene indicate interaction of the two fusion proteins; (+) and (–) signs stand for interaction and no-interaction, respectively.

To identify the domains required for AATF interactions, we constructed a series of plasmids that would express selected regions of ADA2A, ADA2B and GCN5 (Fig 2). The expression of truncated fusion proteins was confirmed by western blotting. Truncations that removed part of the SANT domain of ADA2A and ADA2B or the evolutionarily-conserved C-terminal SWIRM domain did not abolish AATF interaction and for ADA2B, a truncation retaining the SANT domain but lacking both the ‘Ada Box 1’ and ‘Ada Box 2’ and the SWIRM domain also retained interaction with AATF (Fig 2A and 2B). These results suggest that for both ADA2 isoforms, the region between the SANT domain and the Ada1 box also may be critical for interaction with AATF while the SWIRM domain is not required. For hGCN5, amino or carboxy-terminal truncations that removed the acetyltransferase domain no longer manifested interaction with AATF suggesting this domain is required for the association. GCN5 amino terminal truncations retaining only the GCN5 bromodomain failed to assoicate with AATF indicating this region is not sufficient for interaction in the absence of the acetyltransferase domain (Fig 2C). Further carboxy-terminal truncations removing only the bromodomain would be needed to determine whether it is only the acetlytransferase domain that is required for AATF interaction.

### ADA2 and GCN5 proteins co-immunoprecipitate with AATF

**Fig 3:**
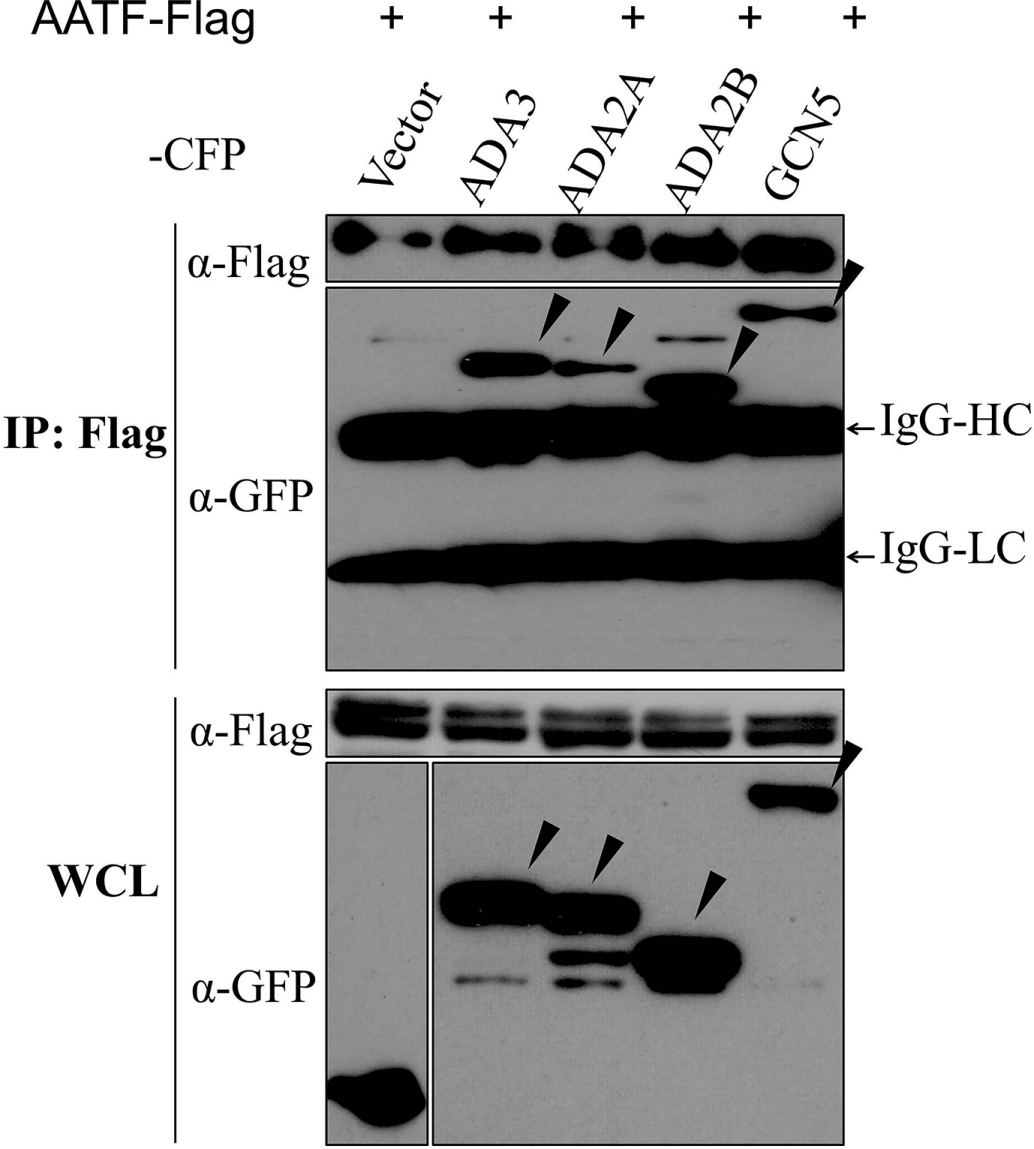
AATF associates with ADA2A, ADA2B and GCN5 proteins in mammalian cells. Protein interactions were detected by co-immunoprecipitations using whole cell lysates (WCL) of HEK293 cells transfected with a plasmid expressing Flag-tagged AATF and a plasmid expressing either CFP (vector) or the indicated CFP fusion protein. Immunoprecipitations (IP) were carried out using monoclonal anti-Flag antibody and precipitated proteins were analyzed by western blotting with monoclonal anti-GFP antibody to detect CFP fusion proteins. IgG-HC and IgG-LC indicate the heavy and light-chain of the primary antibody, respectively. Species with the mobilities expected for the full-length CFP fusion proteins are indicated with arrowheads.

To determine whether the ADA2 and GCN5 interactions with AATF detected in yeast were also detectable in mammalian cells, plasmids encoding a FLAG-epitope-tagged version of AATF (pAATF-FLAG) and CFP-epitope-tagged full-length human ADA2A, ADA2B and GCN5 proteins (pECFP-ADA2A, pECFP-ADA2B and pECFP-GCN5, respectively) were transfected into HEK293 cells and whole cell extracts from transfected cells were analyzed by western blotting (Fig 3). A single major species with a mobility consistent with that expected for the CFP or the CFP fusion protein expressed in the extract was detected with the anti-GFP antibody for each. Fainter lower molecular weight species were also detected in some extracts that might represent either degradation products or premature translational termination products of the target CFP fusion protein that retain the epitope recognized by the antibody.

We then performed co-immunoprecipitation (Co-IP) experiments to identify the *in vivo* relevance of the ADA2 and GCN5 interactions with AATF. The whole cell lysates prepared from co-transfected cells were mixed and subjected to Co-IP using anti-FLAG antibody. We showed that transiently expressed AATF-FLAG was able to precipitate CFP-ADA2A, -ADA2B, -GCN5 but not CFP (Fig 3). The efficiency of immunoprecipitation of the FLAG-tagged AATF seemed somewhat higher in ADA2B and GCN5-transfected cells but this might be a result of variation in the transfection efficiencies of the constructs, which in turn influences the intensities of the bands detected.

### Co-localization of ADA2A, ADA2B and GCN5 with AATF in mammalian cells

**Fig 4:**
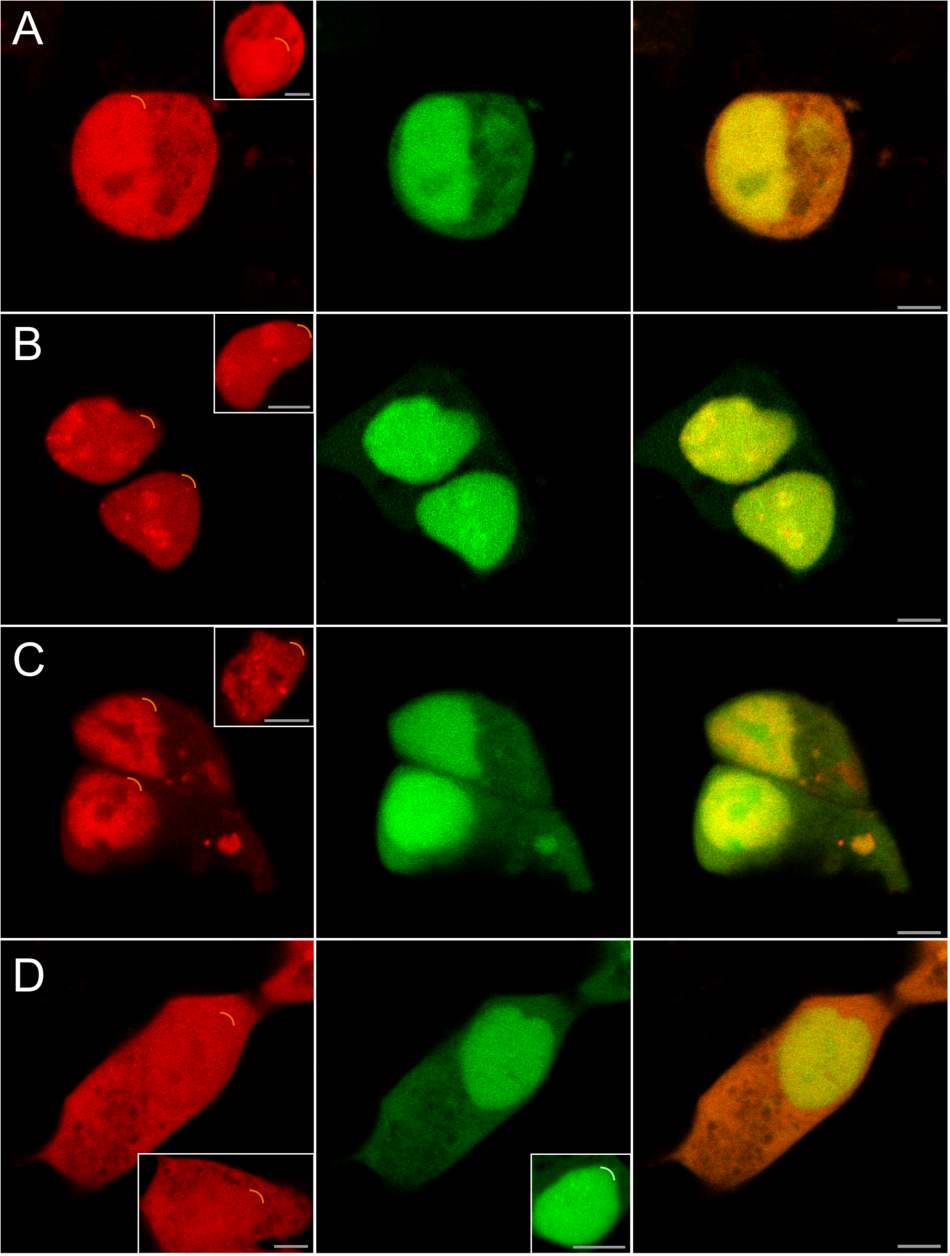
Co-localizations of ADA2A, ADA2B, GCN5 with AATF protein in HEK293 cells. Intracellular co-localization of CFP-conjugated ADA2A (A), ADA2B (B), GCN5 (C), empty vector expressing CFP alone (D) and YFP-conjugated AATF proteins (middle column). Yellow color in the merged image (last column) indicates co-localization. Live cell images were captured by confocal microscopy and pseudo-coloured red (CFP) and green (YFP). Insets show single transfections. Upper right corners of nuclei are marked with a curved line. The last column shows merged images. Scale bar is 5 μm.

To determine whether the physical associations detected in yeast also occurred in mammalian cells, we constructed plasmids that expressed ADA2A, ADA2B and GCN5 as CFP fusion proteins (pECFP-ADA2A, -ADA2B and -GCN5, respectively) and AATF as a YFP fusion protein (pEYFP-AATF). The plasmids were transfected into HEK293 (Fig 4, insets) and U2OS cells (S1 Fig, insets) to first determine the intracellular localization of each in the absence of the AATF YFP fusion. ADA2B (Fig 4B and S1 Fig, insets) and GCN5 (Fig 4C and S1 Fig, insets), as anticipated, were localized in the nucleus, while ADA2A was present in both the nucleus and cytoplasm (Fig 4A and S1 Fig, insets). Unlike the others, ADA2B was also concentrated in nucleolar compartment where the ribosomes are synthesized (Fig 4B and S1 Fig, insets). AATF was mostly found in the nucleoplasm (Fig 4A and S1 Fig, insets). Like ADA2B, the AATF protein, was also localized in the nucleolus in addition to the nucleoplasm (Fig 4 and S1 Fig). Empty vector expressing CFP was used as negative control (Fig 4D and S1 Fig, insets). Consistent with this localization, AATF has been reported to contain two nuclear and two putative nucleolar localization signals and to be mostly nuclear and nucleolar localized [10,11,25].

To examine co-localization of the proteins, HEK293 and U2OS cells were co-tranfected with pECFP-ADA2A, -ADA2B, -GCN5 and pEYFP-AATF and cells were imaged by fluoresence microscopy (Fig 4 and S1 Fig, respectively). The results showed co-localizations of ADA2A with AATF in the nucleoplasm with partial localization in the cytoplasm (Fig 4A and S1 Fig). Distribution of ADA2B and GCN5 was strkingly similar in HEK293 and in U2OS cells. ADA2B co-localization with AATF was primarily in the nucleoplasm with an additional nucleolar co-localization (Fig 4B and S1 Fig). Unlike AATF, GCN5 appeared to be excluded from the nucleolus with co-localization predominantly in the nucleoplasm (Fig 4C and S1 Fig). The co-localization of the proteins in human cells observed by microscopy is consistent with potential physical associations between the partners.

### Transcription activation by AATF is negatively affected by ADA2 and GCN5 expression

**Fig 5:**
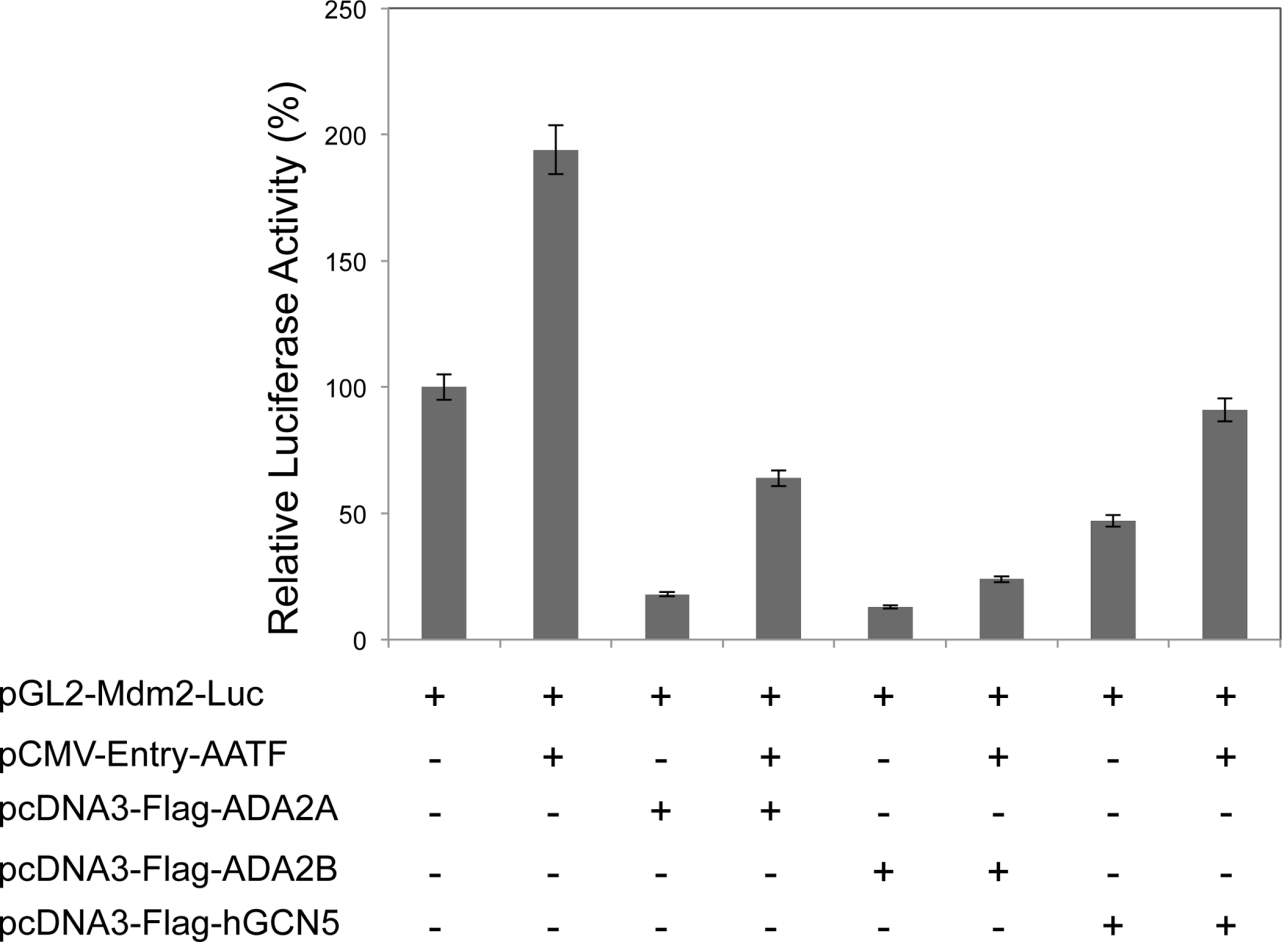
Effect of co-expression of ADA2A, ADA2B, GCN5 with AATF on the expression of an MDM2 promoter-luciferase reporter gene. U2OS cells were transfected with a reporter plasmid encoding a luciferase gene under the control of the MDM2 promoter or co-transfected with the reporter plasmid and plasmids expressing ADA2A, ADA2B, GCN5 with AATF protein partner as indicated. Luciferase activity was measured 48 h post-transfection. Results represent the average ± SD from three independent assays. Co-expression of AATF with ADA2A, ADA2B or GCN5, significantly reduced the activation imparted by AATF on MDM2 promoter-directed transcription (*p* <0.01).

Luciferase assays were conducted to determine whether ADA2 and GCN5 interactions with AATF might influence AATF transcriptional activation function. U2OS cells were transfected with a luciferase reporter gene under the control of a promoter previously reported to be activated by AATF, the MDM2 promoter [9]. We used the constructs pCMV-Entry-AATF, pcDNA3-FLAG-hADA2A, pcDNA3-FLAG-hADA2B and pcDNA3-FLAG-hGCN5 in order to ensure transient expression of the specific proteins and pMDM2-luc as reporter. A plasmid directing the expression of AATF was transfected into U2OS cells together with ADA2A, ADA2B or GCN5-encoding mammalian expression vectors and luciferase activity was measured using a luminometer (Fig 5). Our results showed that as previously reported [9], AATF expression activates the promoter while co-expression of ADA2A, ADA2B or GCN5 with AATF, significantly reduced the activation imparted by AATF on MDM2 promoter-directed transcription (*p* <0.01) (Fig 5).

## Discussion

The yeast hybrid technique has frequently been used to identify protein-protein interactions [23]. Based on our previous Y2H study reporting four new partners of ADA3 we hypothesized the other components of ADA3-containing HAT complexes, ADA2 and GCN5 could also be partners of ADA3-interacting proteins [9]. The rationale behind the hypothesis is based on the known involvement of ADA3 with the other HAT components in control of gene expression. In this study we showed AATF as a novel interacting partner of ADA2 and GCN5.

Since ADA2 isoforms, ADA2A and ADA2B are found in distinct complexes differing both in size and subunit compositions, which could, in turn reveal different interactions, we included both variants in our investigation. Indeed, the two-hybrid interaction of ADA2B with AATF, more strongly activated the reporter genes than the interaction with ADA2A. Although the degree of interaction in this assay may not correlate with the strength of interactions between ADA2 variants in the native context, it may be noteworthy, because the metazoan ATAC and SAGA are distinct multi-subunit co-activators responsible for dynamic reversible acetylation of histones. Both the SAGA and ATAC complexes contain a HAT module composed of the acetyltransferase enzyme GCN5/PCAF as well as several ADA family proteins that were suggested to modulate the catalytic activity of the HAT enzyme [26–28]. Although the localization sites of ATAC and SAGA overlap to some extent, the former is recruited to enhancers and promoters while SAGA is mainly recruited to promoters [29]. While they share similarities in protein composition and organization, the complexes are functionally distinct. Therefore, our data is well in accordance with the literature reports because different levels of AATF interactions to ADA2A and ADA2B could be attributed to their specific biological roles.

Our results showed that AATF interacts with three known components of HAT complexes GCN5, ADA2 and ADA3 [9] but we did not observe interaction between AATF and SGF29, a subunit of SAGA complexes. Interaction in yeast in the two-hybrid assay may not have been detected if the presence of human ADA2 and GCN5 proteins is required to bridge AATF association with SGF29 or might indicate that AATF interacts only with the ADA complexes that lack SGF29.

We determined that the evolutionarly-conserved SWIRM domain of both isoforms of ADA2 was not required for interaction with AATF and that for ADA2B, the amino-terminal portion of the protein containing the SANT domain was both sufficient and required for this interaction. For GCN5, our truncation analysis indicated that residues between 428 and 533 which includes part of the ‘Acetyltransferase’ domain were required. Further truncations are needed to determine whether the acetyltransferase domain is sufficient for interaction of if sequences immediately amino-terminal to this domain are also involved. The C-terminal ‘Bromodomain’ of GCN5, although not shown to be required, was found not to be sufficient for AATF interaction in the absence of the acetyltransferase domain.

ADA2 isoforms bind to GCN5 via their N-termini and the N-terminal proximal central regions are important for the GCN5 interaction [30]. The SWIRM domain of ADA2 proteins, on the other hand, is required for binding to double stranded DNA and access of the protein to nucleosomal histones [31]. Therefore, this domain could rather be required for DNA-protein interactions that are important in chromatin remodeling. Moreover, it was reported that C-terminal regions of ADA2 proteins play an important role in the incorporation of ADA2 into SAGA- or ATAC-type complexes and determine H3- or H4-specific histone targeting [32]. The acetyltransferase domain, which we report as region GCN5 required for its interaction with AATF, is essential for GCN5 functions. This region participates in the control of transcriptional activation via acetylation of lysine residues of histone and non-histone proteins [33]. Finally, our results suggest that the GCN5 bromodomain, found in chromatin-related proteins bearing HAT activity which mediates the interactions with acetylated lysine residues was not sufficient to for GCN5 interaction with AATF in the absence of the acetyltransferase domain, although our analysis did not establish if the bromodomain was dispensable for the interaction. Therefore, our findings on the mapping of the interactions are strongly supported by prior literature reports related to the functions of these domains.

We were also able to demonstrate co-immunoprecipitation of ADA2A, ADA2B and GCN5 with AATF in mammalian cells (Fig 3). Microscopy analyses demonstrated that co-localization of the proteins in human cells were consistent with potential physical associations between the partners. Furthermore we examined the effect of the interactions on transcriptional activation of partners using reporter gene assays. We showed that ADA2s and GCN5 negatively affect transcription activation by AATF. The effects on the promoter of the ADA2 and GCN5 could be independent, i.e., each of the partners may lead to the loss of activation while AATF increases.

Our data is important to further elucidate tumorigenesis and DNA repair pathways. The tumor suppressor p53 is a key component of DNA damage response. However, the signaling molecules that dictate choice among different cellular outcomes remain incompletely understood. AATF functions in MAPK signal transduction pathway and in p53-dependent apoptosis [34]. AATF may be particularly significant as a therapeutic target since it selectively represses p53-driven apoptosis in a phospho-dependent manner by preventing the induction of the p53 target genes [34]. ADA3 directly binds and acetylates p53, reducing Mdm2-mediated ubiquitylation of p53 and subsequently stabilizes p53 expression and elevates transcriptional activity of the genes that are the targets of p53 [35,36]. On the other hand, GCN5 is recruited to p53-dependent promoters, a function also attributed to ADA2B, but not to ADA2A [28]. Bennett and Peterson described a model suggesting that NuA4 and GCN5 were both required for the recruitment of SWI/SNF in response to a double strand break, which in turn promotes H2A.X phosphorylation by the checkpoint kinases Mec1 and Tel1 [37]. Moreover, it is reported that acetylation signaling has an important role in regulating the DDR and maintaining genome integrity [38].

Our findings may provide new insights into AATF-mediated transcriptional regulation. We previously reported that Che-1/AATF associated with HAT complexes through its interaction with the transcriptional coactivator ADA3 [9]. In this study, we showed that this interaction is extended to both isoforms of ADA2, ADA2A and ADA2B and to GCN5, other subunits of the HAT domain of different complexes. Based on these observations, one could argue that Che-1/AATF could be participating in the regulation of gene expression by regulating chromatin structure. Che-1/AATF is post-translationally modified and these modifications affect its function, localization and its interactions [13,15]. Since HAT complexes acetylate histone and non-histone proteins, Che-1/AATF could be modified by HAT complexes. Furthermore, Che-1/AATF has been identified as a nucleolar stress sensor [39]. Our observations showed that hADA2B co-localization with AATF was primarily in the nucleoplasm with an additional nucleolar co-localization. In this context, it would be interesting to further investigate the possibility of ADA2B involvement in the nucleolar stress response that has a crucial role in maintaining cellular homeostasis. In the last few years the nucleolus and ribosomal gene expression have emerged as new targets for cancer therapy and RNA polymerase I inhibitors are currently entering phase I clinical trials [40]. Future efforts focused on understanding the mechanism of action of Che-1/AATF are needed to fully understand its functions and to establish its potential as a therapeutic target.

Taken together, our data suggest AATF interaction is not limited only to ADA3 but extends to other components of the ADA3-containing HAT complexes. Further systematic investigations will shed light on identifying additional aspects of the function of multi-faceted chromatin-associated complexes.

## Materials and methods

### Plasmid constructions

Yeast expression plasmid encoding AATF on GAL4 activation domain (Gal4_AD_) vector was constructed by subcloning *Sal*I+*Not*I fragment from pCMV-Entry-AATF (Origene, Rockville, MD, USA) into pGADT7 vector (Clontech, Mountain View, CA, USA). Open Reading Frames (ORF) for *ADA2A*, *ADA2B*, *GCN5 and SGF29* genes, comprised of the cDNAs of 1332 bp, 1263 bp, 2514 bp and 882 bp, respectively, were amplified by Polymerase Chain Reaction (PCR) (see Table 1 for primers). Amplified regions were cloned into pGBKT7 GAL4 binding domain (Gal4_BD_) vector (Clontech, Mountain View, CA, USA) using *Bam*HI and *Pst*I, *Bam*HI and *Eco*RI enzyme pairs for ADA2A and ADA2B, respectively and *Eco*RI and *Sal*I enzyme pairs for GCN5 and SGF29, respectively.

**Table 1:** The sequences of the primers used in the amplification of corresponding genes.

Primer Name	Primer Sequences (5’-3’)
**ADA2A-pGBKT7**	F: CGGCGGATCCGAATGGACCGTTT R: CATACTGCAGGGAGCCTTAGCCTTTA
**ADA2B-pGBKT7**	F: CGGCGAATTCAAGATGGCGGAGCTGGGGAA R: CATAGGATCCAGCTTCAAGACGCGT
**GCN5-pGBKT7**	F: CGGCGAATTCGCCATGGCGGAACCTT R: CATAGTCGACGGCCTACTTGTCAAT
**ADA2A-N1**	F: CGGCGGATCCACCTCATGGAGCCTT
**ADA2A-N2**	F: CGGCGGATCCCTGTGATGGACTGT
**ADA2A-N3**	F: CGGCGGATCCGGTACATGCCAGCT
**ADA2A-N4**	F: CGGCGGATCCAATTAATGGAACGGC
**ADA2A-N5**	F: CGGCGGATCCGCACTATGCTCTCA
**ADA2A-N6**	F: CGGCGGATCCTTCCAATGGCTTCGA
**ADA2A-C1**	F: CGGCGGATCCGAATGGACCGTTT
**ADA2B-N1**	F: CGGCGAATTCGAAGATATGGCTGCC
**ADA2B-N2**	F: CGGCGAATTCGGCTACATGCCGCT
**ADA2B-N3**	F: CGGCGAATTCGTGGACATGTACGT
**ADA2B-N4**	F: CGGCGAATTCCAGTTCATGTCAT
**GCN5-N1**	F: CGGCGAATTCCGCATGGATCTGCA
**GCN5-N2**	F: CGGCGAATTCCAGATGACCCGGCCT
**GCN5-N3**	F: CGGCGAATTCTCCATGCTGGAGGAGGAGAT
**GCN5-N4**	F: CGGCGAATTCGAGCCTATGCCAGGC
**GCN5-N5**	F: CGGCGAATTCCCGCGCATGCCTAA
**GCN5-N6**	F: CGGCGAATTCACGCTGATGGAGT
**GCN5-N7**	F: CGGCGAATTCAAGACCATGACTGA
**SGF29-pGBKT7**	F: CATAGAATTCACAATGGCCCTCGTGTCT R: CGGCGTCGACGCATCACTTTTTCTTGGGT

To map the region/regions of interaction we constructed N- and C-termini truncated versions of ADA2A, ADA2B and GCN5 plasmids. Deletions at N-termini were generated by PCR (See Table 1 for primer sequences) using full length pGBKT7-ADA2A, -ADA2B and -GCN5 plasmid DNAs as templates while the cDNAs encoding the proteins of interest with altered C-termini were created by subcloning *Stu*I+*Bam*HI, *Kpn*I+*Bam*HI, *Sac*I+*Bam*HI fragments from pGBKT7-ADA2B and *Bgl*II+*Sal*I, *Sac*II+*Sa*lI, *Bam*HI+*Sal*I fragments from pGBKT7-GCN5 into pGBKT7. C-termini truncated versions of ADA2A were generated by PCR (See Table 1 for primer sequences).

The plasmids expressing ADA2A, ADA2B, GCN5 as cyan fluorescent protein (CFP) fusion proteins were created by digesting pGBKT7-ADA2A, -ADA2B and -GCN5 constructs using *Bgl*II and *Pst*I, *Eco*RI and *Bam*HI, *Eco*RI and *Sal*I restriction enzyme pairs, respectively, to excise ORF of ADA2B, GCN5 and ADA2A. The fragments were then ligated with pECFP-C2 and pECFP-N1 plasmids, respectively, digested with the same enzymes. Plasmid expressing the AATF-fused to yellow fluorescent protein was constructed as described before [9]. All PCR-amplified genes were checked after cloning by sequencing and the constructs were verified via restrictional digestions. All the restriction enzymes used for restrictional control of the constructs were from NEB (Ipswich, MA, USA).

pcDNA3-FLAG-ADA2A, -ADA2B and -GCN5 constructs were kindly provided by Dr. Imre Boros (Institute of Biochemistry, Hungarian Academy of Sciences) and Dr. László Tora (IGBMC, University of Strasbourg).

### Yeast strains and media

Two-hybrid reporter yeast strains used in this study were *Saccharomyces cerevisiae (S*. *cerevisiae*) AH109 (*MATa*, *trp1-901*, *leu2-3*, *112*, *ura3-52*, *his3-200*, *gal4*Δ, *gal80*Δ, *LYS2*::*GAL1*_*UAS*_ *GAL1*_*TATA*_*-HIS3*,*GAL2*_*UAS*_*-GAL2*_*TATA*_*-ADE2*, *URA3*::*MEL1*_*UAS*_*-MEL1*_*TATA*_*-lacZ*, *MEL1)* (Clontech, Mountain View, CA, USA). Yeast cells were cultured at 28°C in YEPD + adenine (1% yeast extract, 2% bactopeptone, 2% dextrose, 1% adenine) or SD medium (0.67% yeast nitrogen base, 2% dextrose, supplemented with appropriate amino acids and bases) [41]. Media containing 40 μg/mL X-alpha-gal (Clontech, Mountain View, CA, USA) were solidified with 20% agar.

### Yeast two-hybrid assay

The GAL_4_-BD and -AD constructs bearing the genes of interest were transformed into yeast AH109 strain for expression, toxicity and auto-activation experiments before Y2H assay as previously described [9]. The expression, toxicity and auto-activation experiments were also performed for truncated/deleted versions of ADA2A, ADA2B and GCN5 constructs for testing against AATF interactions.

The Y2H experiments were performed as described before [9,42]. Briefly, to test potential protein–protein interactions, co-transformants were screened for growth in a selective medium lacking adenine, histidine, leucine, tryptophan but in the presence of X-α-gal (SD/-Ade/-His/-Leu/-Trp/X-α-gal) to assess expression of the *HIS3*, *ADE2*, *MEL1* and *LacZ* reporter genes under the control of the Gal4-responsive Upstream Activating Sequence (UAS).

### ß-Galactosidase liquid assay

Quantitative analyses of *β-gal* activity were performed using O-nitrophenyl β-D-galactosidase (ONPG) chromogenic substrate in triplicate as previously described [24,42].

#### Mammalian cell culture and transfection

U2OS and HEK293 cells were cultured in Dulbecco's modified Eagle medium containing 10% fetal bovine serum and 1% penicillin–streptomycin. Cells were grown in an incubator at 37°C with 5% CO_2_. Transient plasmid transfections were performed using polyethyleneimine (PEI) reagent (Sigma-Aldrich, Milwaukee, WI, USA) according to the standard protocol or Turbofect transfection reagent (Thermo, Lithuania) according to the manufacturer’s procedure [43,44]. Reagents used for cell culture were purchased from Invitrogen (Carlsbad, CA, USA).

### Western blot analysis

For expression analyses, proteins were extracted from yeast by alkaline lysis method as described [45]. Briefly, yeast cells were grown on liquid medium to an OD_600_ of 1–2. After harvesting cells by centrifugation, the cells were resuspended in 200 μL of lysis solution (1.85 M NaOH, 7.4% β-mercaptoethanol) and incubated on ice for 10 min. Protein was precipitated by addition of 200 μL ice cold 50% trichloroacetic acid (TCA), pelleted by centrifugation, washed twice with 1 mL ice cold acetone, dried and resuspended in appropriate amount of 2x protein gel loading buffer (125 mM Tris pH 6.8, 4.0% SDS, 20% glycerol, 4.0% β-mercaptoethanol, 1 M urea, 0.05% bromophenol blue, 0.05% xylene cyanol). Western blot analyses were performed using anti-Myc mouse monoclonal antibody (Cell Signaling Technology, Danvers, MA, USA) for Myc-epitope tagged Gal4_BD_-fusions from pGBKT7-based plasmids.

For expression of FLAG epitope-tagged ADA2, ADA3, GCN5 and AATF; CFP epitope-tagged ADA3, ADA2 and GCN5; YFP epitope-tagged AATF proteins in mammalian cells, the fusion proteins extracted from the cells and were subjected to western blot analysis using an anti-FLAG M2 (Sigma-Aldrich, Milwaukee, WI, USA) and anti-GFP antibodies (Proteintech Group, Rosemont, IL, USA), respectively.

### Co-immunoprecipitation assay

For Co-IP analyses HEK293 cells were seeded in 10 cm petri dishes (≈5x10^6^ cell/dish) in complete DMEM 18 h prior to transfection and transiently transfected/co-transfected with 6 μg of pECFP-ADA3, pECFP-ADA2A, pECFP-ADA2B, pECFP-GCN5 and pCMV-Entry-AATF DNAs using PEI transfection reagent according to the standard procedure [44]. Media were removed and replaced with fresh complete DMEM 6 h post-transfection. Following 40 h of incubation, cells were scraped and harvested by centrifugation at 2000 rpm for 5 min, the supernatant was removed and the pellet was washed with phosphate-buffered saline (PBS), and lysed in IP buffer (25 mM HEPES, pH 7.9, 150 mM KCl, 0.1% Nonidet P-40, 1 mM EDTA, 1 mM dithiothreitol, 10% glycerol, 0.1 mM phenylmethylsulfonyl fluoride, 10 g/ml aprotinin, 10 g/ml leupeptin, and 1 g/ml pepstatin A). After rotation of lysates for 1 h at 4°C, the cell debris was removed by centrifugation at 13500 rpm for 10 min and the supernatant was used as the whole cell extract. In each IP experiment, approximately 0.5–1 mg of purified proteins were incubated with anti-FLAG M2 (Sigma-Aldrich, Milwaukee, WI, USA) in IP buffer by rotating at 4°C for overnight. Immunoprecipitates were washed for four times with ice-cold IP buffer and the beads were collected by centrifugation at 2000 rpm for 2 min at 4°C. Bound proteins were eluted in 2x protein gel loading buffer, subjected to SDS–PAGE and transferred onto a PVDF membrane. Coprecipitated proteins were detected by western blot analysis using an anti-GFP antibody (Proteintech Group, Rosemont, IL, USA) with the enhanced chemiluminescence (ECL) kit (Bio-Rad, Hercules, California, USA).

### Co-localization analyses

Human U2OS and HEK293 cells were seeded in 8-well chamber slides (1x10^4^ cell/well) in complete DMEM and transfected next day with 0.25 μg of CFP and YFP fusion plasmids using Turbofect transfection reagent (Fermentas, Lithuania) as previously described [9]. Confocal laser scanning microscopy was performed using Olympus Fluoview FV1000 laser scanning microscope (Olympus Life Science Europa GmbH, Hamburg, Germany). Microscope configuration was the following: objective lens UPLSAPO 40x (oil, NA 1.3); sampling speed: 8μs/pixel; scanning mode: sequential unidirectional; excitation: 458 nm (CFP) and 515 nm (YFP); laser transmissivity: 30% and 10% were used for CFP and YFP, respectively; main dichroic beamsplitter: DM458/515, intermediate dichroic beamsplitter: SDM 510; CFP was detected between 470–520 nm and YFP was detected between 520–570 nm. Differential interference contrast (DIC) or standard transmission images were captured with 515 nm laser line. Composite images were prepared using CorelDraw Graphics Suite X7 (Corel).

### Luciferase reporter gene assay

For luciferase reporter assays U2OS cells were transfected with 0.2 μg of MDM2 promoter-luciferase plasmid (pGL2-MDM2-Luc) using Turbofect transfection reagent (Fermentas, Lithuania) according to manufacturer’s protocol. We then transfected the cells having the MDM2 promoter reporter with either individual pcDNA3-FLAG-ADA2A, -ADA2B and -GCN5 constructs (0.2 μg) or co-tranfected these with the plasmid encoding AATF. Cells were lysed at 24 h post-transfection and assayed for luciferase activity using the luciferase reporter assay system (Promega, Madison, WI, USA) as described [9]. The luciferase activity in each sample was manifested by adding 20 μL of luciferin to 20 μL of each cell lysate and relative luciferase activity from three independent experiments was measured using the Luciferase Assay system (Berthold Detection System Orion L/MPL4, Pforzheim, Germany) according to the manufacturer’s guidelines.

### Statistics

All data were presented as mean (±SD) and processed with the SPSS v 11.0 statistical software package. One-way analysis of variance (ANOVA) followed by Bonferroni adjustment was used for multiple comparisons of data presented in Figs 1C and 5. The level of statistical significance was set at *p*<0.05.

## Supporting information

S1 Fig Co-localizations of ADA2A, ADA2B, GCN5 with AATF protein in U2OS cells. U2OS cells were co-transfected with two plasmids, one expressing CFP-conjugated ADA2A (A), ADA2B (B), GCN5 (C), or an empty vector expressing CFP alone (D), and a second plasmid that encoded YFP-conjugated AATF (middle column). Yellow color in the merged image (last column) indicates co-localization. Live cell images were captured by confocal microscopy and pseudo-coloured red (CFP) and green (YFP). Insets show single transfections. Upper right corners of nuclei are marked with a curved line. Last column shows merged images. Scale bar is 5 μm.
(DOCX)
