# Supplementary material for: Che1/AATF interacts with subunits of the histone acetyltransferase core module of SAGA complexes
Source: PLoS One. 2017 Dec 12;12(12):e0189193. doi: 10.1371/journal.pone.0189193 (PMC5726650; doi:10.1371/journal.pone.0189193)

**Supporting Information for**

**Che1/AATF interacts with subunits of the histone acetyltransferase core module of SAGA complexes**

**Gizem Caliskan^1,^ ^¶^, Ikbal C. Baris^2, ¶^, Ferhan Ayaydin^3^, Melanie J. Dobson^4^, Muge Senarisoy^1^, Imre M. Boros^5^, Zeki Topcu^1^, Sevil Zencir^2,^***

^1^Department of Pharmaceutical Biotechnology, Faculty of Pharmacy, Ege University, Izmir, Turkey

^2^Department of Medical Biology, Faculty of Medicine, Pamukkale University, Denizli, Turkey

^3^Cellular Imaging Laboratory, Biological Research Center, Hungarian Academy of Sciences, Szeged, Hungary

^4^Department of Biochemistry & Molecular Biology, Dalhousie University, Halifax, NS, Canada

^5^Institute of Biochemistry, Hungarian Academy of Sciences, Szeged, Hungary

^¶^These authors contributed equally.

***** Corresponding author:

E-mail: [sevilz@pau.edu.tr](mailto:sevilz@pau.edu.tr).

**Fig S1. Co-localizations of ADA2A, ADA2B, GCN5 with AATF protein in U2OS cells**. U2OS cells were co-transfected with two plasmids, one expressing CFP-conjugated ADA2A (A), ADA2B (B), GCN5 (C), or an empty vector expressing CFP alone (D), and a second plasmid that encoded YFP-conjugated AATF (middle column). Yellow color in the merged image (last column) indicates co-localization. Live cell images were captured by confocal microscopy and pseudo-coloured red (CFP) and green (YFP). Insets show single transfections. Upper right corners of nuclei are marked with a curved line. Last column shows merged images. Scale bar is 5 μm.


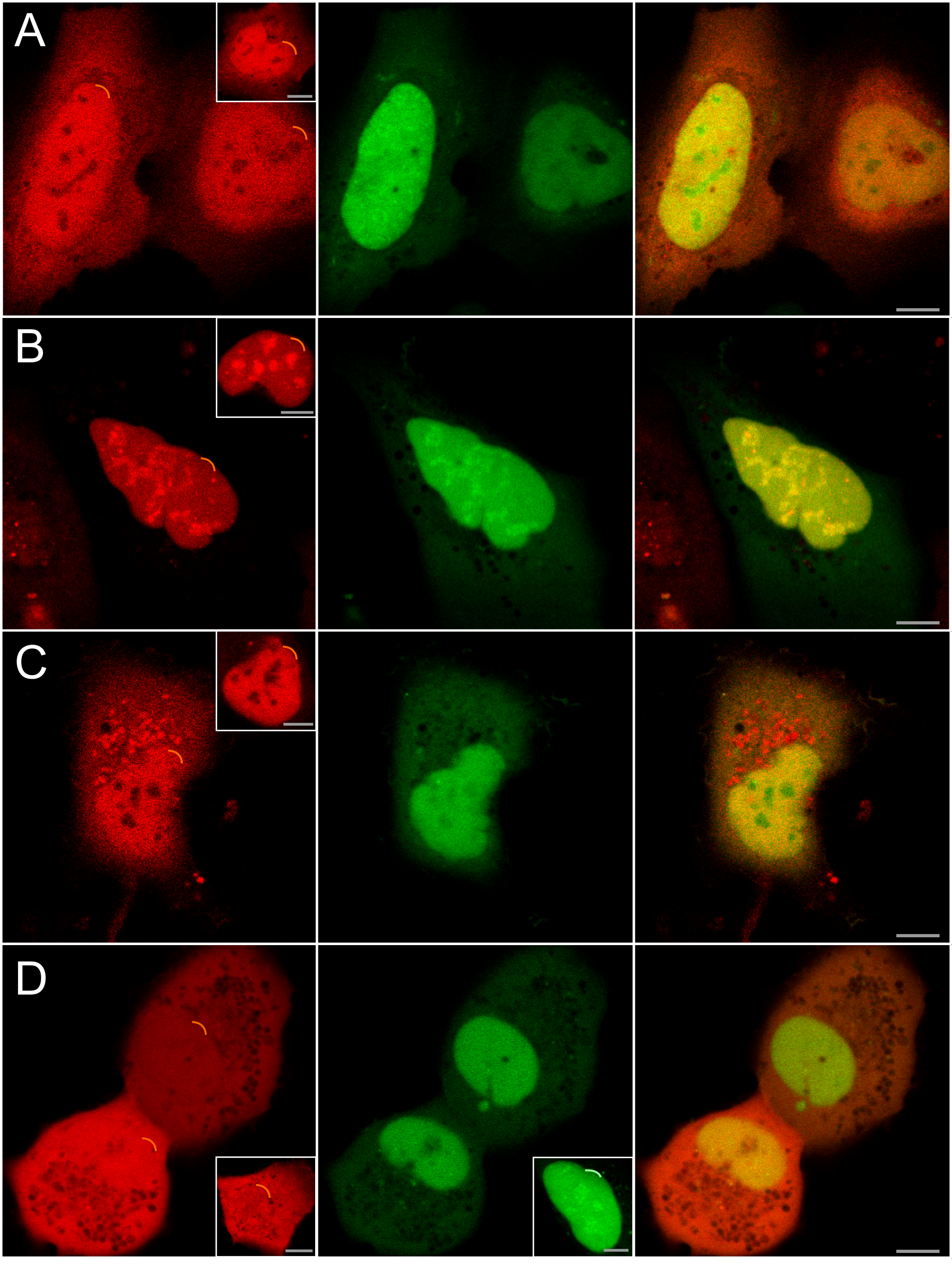

Supplement: S1 Fig — U2OS cells were co-transfected with two plasmids, one expressing CFP-conjugated ADA2A (A), ADA2B (B), GCN5 (C), or an empty vector expressing CFP alone (D), and a second plasmid that encoded YFP-conjugated AATF (middle column). Yellow color in the merged image (last column) indicates co-localization. Live cell images were captured by confocal microscopy and pseudo-coloured red (CFP) and green (YFP). Insets show single transfections. Upper right corners of nuclei are marked with a curved line. Last column shows merged images. Scale bar is 5 μm. (DOCX) [file pone.0189193.s001.docx]
